# Relative Accuracy of Cervical and Anal Cytology for Detection of High Grade Lesions by Colposcope Guided Biopsy: A Cut-Point Meta-Analytic Comparison

**DOI:** 10.1371/journal.pone.0038956

**Published:** 2012-07-25

**Authors:** Edward R. Cachay, Wollelaw Agmas, William C. Mathews

**Affiliations:** Department of Medicine, University of California at San Diego, San Diego, California, United States of America; The Chinese University of Hong Kong, Hong Kong

## Abstract

**Background:**

We recently reported, using a receiver operating characteristic area metric, the first meta-analytic comparison of the relative accuracy of cervical and anal cytology in detecting moderate or severe histopathologic lesions by magnification directed punch biopsy. The aim of the present research was to meta-analytically examine cut-point specific operating characteristics (sensitivity, specificity) of cervical and anal cytology in detecting high grade squamous intraepithelial lesion (HSIL) histopathology by colposcope directed punch biopsy.

**Methodology/Principal Findings:**

The primary eligibility requirement was availability of tabulated cytology (normal, atypical cells of unclear significance [ASCUS], low grade squamous intraepithelial lesion, HSIL or atypical squamous cells cannot rule out high grade [ASC-H]) and biopsy (<HSIL, ≥ HSIL) counts. Meta-analysis and meta-regression of diagnostic accuracy was performed with examination of study quality criteria and heterogeneity. Thirty-three cervical and 11 anal publications were eligible between 1990 and 2010. Meta-analytically cut-point analysis showed that using a cut-point of ASCUS the sensitivity in both settings is similar while anal cytology is less specific than cervical cytology (specificity [95% confidence interval] 0.33 [0.20–0.49] vs. 0.53[0.40–0.66], p = 0.04) for the detection of HSIL histopathology by colposcope directed punch biopsy.

**Conclusions/Significance:**

Using a cytology cut-point of HSIL or ASC-H, anal cytology is less sensitive but comparably specific to cervical cytology. However, using a cut-point of ASCUS, differences in accuracy were of borderline significance.

## Introduction

In response to increasing rates of invasive anal cancer among HIV-infected persons [1], [2], a growing number of HIV clinics are implementing screening programs for anal cancer and its precursors modeled on procedures used in cervical cancer screening [3]. We recently reported, using a *receiver operating characteristic (ROC)* area metric, the first meta-analytic comparison of the relative accuracy of cervical and anal cytology in detecting moderate or severe (high grade squamous intraepithelial lesion -HSIL) histopathologic lesions by magnification directed punch biopsy [4]. While ROC area is a useful summary measure of test discrimination, a more clinically useful metric would be based on test sensitivity and specificity at varying cytology cut-points. We therefore conducted a secondary meta-analytic comparison of the previously published summary data tables. The aim of the present research was to meta-analytically examine *cut-point specific operating characteristics* (sensitivity [SE], specificity [SP]) of cervical and anal cytology in detecting HSIL histopathology by colposcopic and high resolution anoscopic (HRA) directed punch biopsy.

## Methods

Eligible studies were identified by MEDLINE citation of relevant publications between 1990 and 2010 published in the English literature as described in flowchart of included studies in our original publication [4]. Briefly, the *index test* was cytologic sampling of cervicovaginal or anal canal tissues. The *reference standard* was defined as colposcope magnified and directed punch biopsy of the uterine cervix or anal canal, respectively. The addition of endocervical curettage sampling was allowed for colposcopy studies. Cytology diagnostic categories include negative (“no atypical or malignant cells” [NAMC]), atypical squamous cells of uncertain significance (ASCUS), atypical squamous cells can’t rule out high grade (ASC-H), low grade squamous intraepithelial lesion (LSIL), and HSIL. “Cases” are defined as those with histopathologic evidence of HSIL or greater by colposcope directed punch biopsy. Cases included cervical or anal intraepithelial neoplasia 2 (CIN 2 or AIN 2), CIN/AIN 3, Carcinoma in situ or invasive carcinoma. A primary study eligibility requirement was availability of cross- tabulated cytology (normal, ASCUS, LSIL, HSIL or ASC-H) and biopsy (<HSIL, ≥ HSIL) counts. Source study quality was rated using QUADAS criteria as previously published [4].

**Figure 1:**
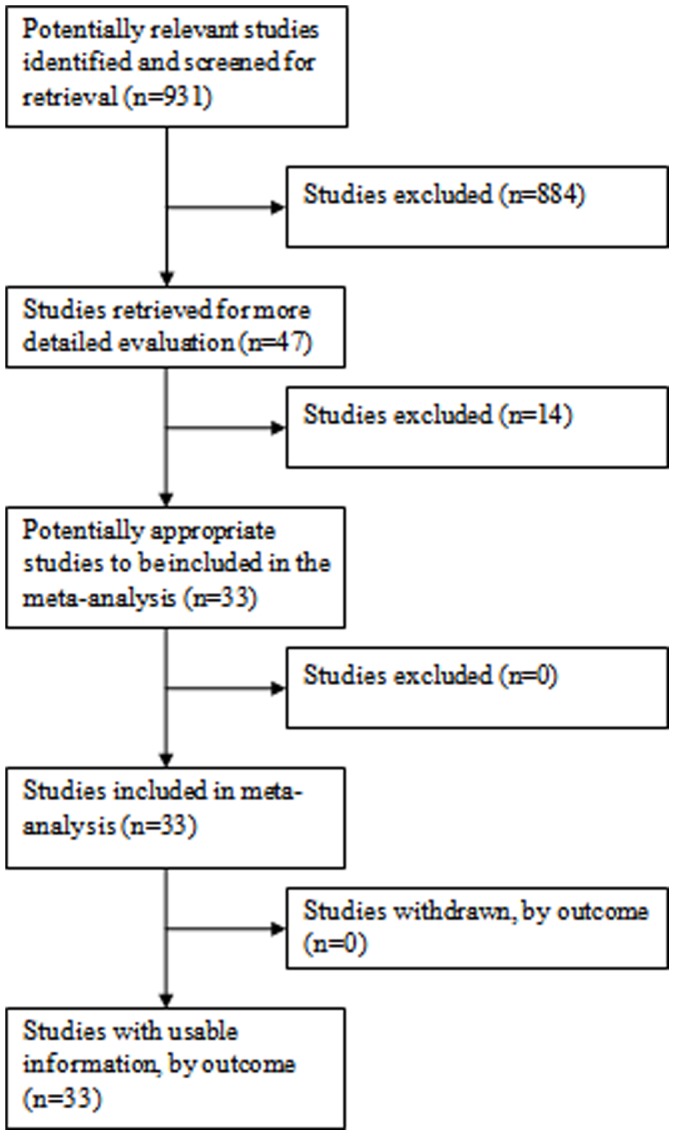
Flow of Included Studies: Cervical Screening.

**Figure 2:**
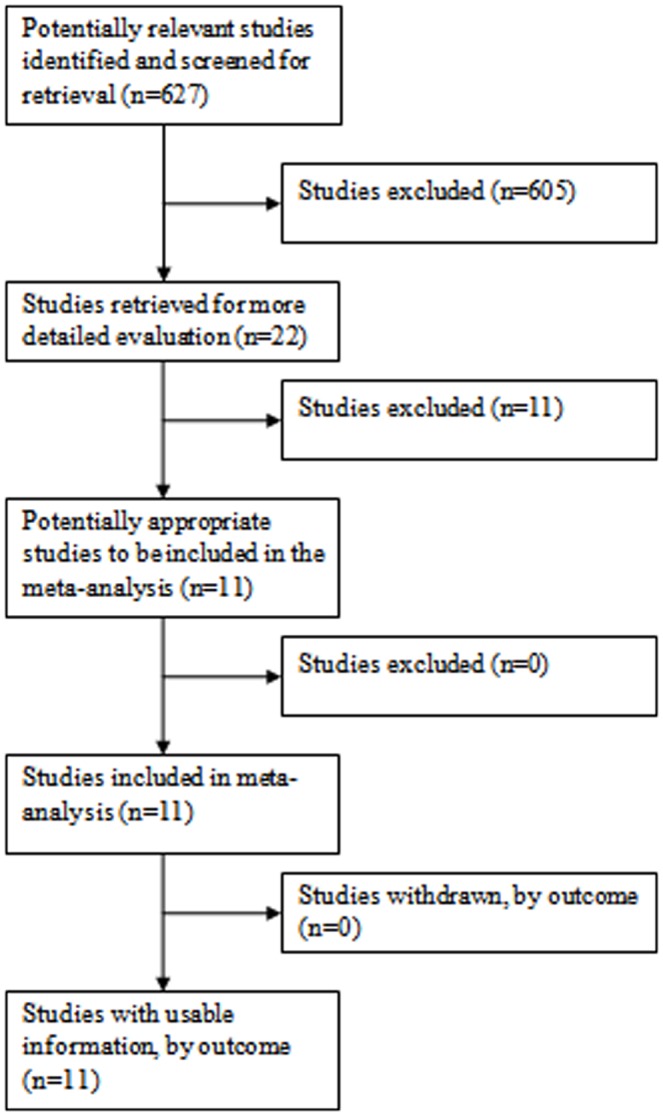
Flow of Included Studies: Anal Screening.

**Table 1:** Meta-analytically cut-point comparison of the joint sensitivity and specificity of cervical and anal cytology for biopsy confirmed high grade dysplasia.

	Sensitivity (SE)				Specificity (SP)			
Cytology Cut-Point	Anal		Cervical		Anal		Cervical	
	SE	(95% CI)	SE	(95% CI)	SP	(95% CI)	SP	(95% CI)
**(HSIL or ASC-H) vs. (LSIL, ASCUS, Normal)^1^**	0.30	(0.19–0.44)	0.63	(0.56–0.69)	0.93	(0.90–0.95)	0.96	(0.95–0.98)
**(HSIL or ASC-H, LSIL) vs. (ASCUS, Normal)^2^**	0.73	(0.62–0.82)	0.80	(0.75–0.85)	0.55	(0.45–0.65)	0.76	(0.66–0.83)
**(HSIL or ASC-H, LSIL, ASCUS) vs. (Normal)^3^**	0.90	(0.76–0.96)	0.91	(0.88–0.94)	0.33	(0.20–0.49)	0.53	(0.40–0.66)

1. Joint model comparison (cervical vs. anal): p<0.001; I^2^ = 92.

2. Joint model comparison (cervical vs. anal): p<0.001; I^2^ = 82.

3. Joint model comparison (cervical vs. anal: p = 0.04; I^2^ = 68.

CI =  Confidence Interval, HSIL =  High grade squamous intraepithelial lesion, ASC-H =  Atypical squamous cells can’t rule out high grade, LSIL =  Low grade squamous intraepithelial lesion, ASCUS =  Atypical squamous cells of uncertain significance.

**Figure 3:**
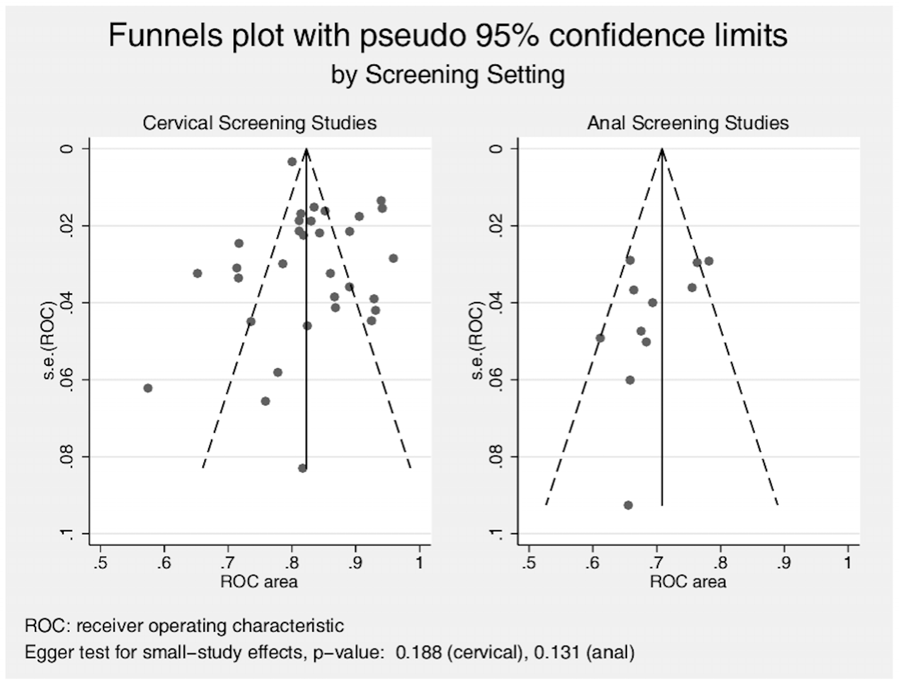
Funnel plots with pseudo 95% confidence limits, by Screening Setting.

**Figure 4:**
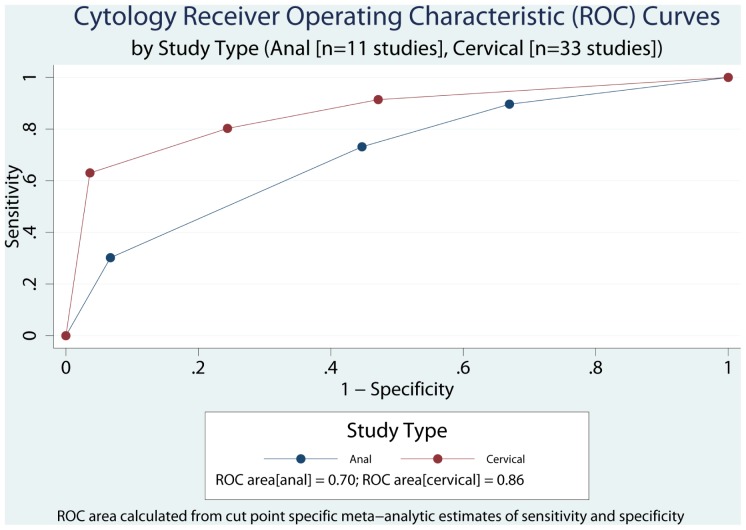
Performance of Diagnostic Receiver Operating Characteristic (ROC) areas by Screening Setting (Cervical, Anal) at different cut-points for identification of High grade squamous intraepithelial lesion histological lesions.

We meta-analytically compared the joint sensitivity and specificity of cervical and anal cytology for biopsy confirmed high grade dysplasia or carcinoma. In this analysis, cytology as the index test was treated as a four-level ordinal measure (NAMC, ASCUS, LSIL, ASC-H or HSIL or carcinoma) and histopathology as the reference standard, as a dichotomous outcome ( <HSIL, ≥ HSIL). Meta-analysis and meta-regression of diagnostic accuracy was performed using *metandi* and *midas*, respectively, implemented in Stata 11.2. Heterogeneity was examined using the I^2^statistic [5]. To illustrate the cut-point dependent tradeoffs between sensitivity and specificity, we plotted the ROC curves for anal and cervical cytology and calculated the corresponding ROC areas using the trapezoidal rule implemented using the *integ* function in Stata [6].

## Results

Thirty-three cervical and 11 anal publications were eligible according to the MEDLINE search algorithm and evaluation of review papers, Figures 1 and 2. Table S1 (supplementary information) presents the data extraction results and summary metric (cytology-biopsy ROC area) organized by study type (cervical and anal [7]–[50]. Table 1 presents the principal meta-analytic comparisons of the ability of cervical and anal cytology to differentiate between high grade and non-high grade histology by colposcope directed biopsy of uterine cervix and anal canal respectively, using different cytology cut-points. Using a cytology cut-point of either HSIL or ASC-H, cervical cytology, compared to anal cytology, had better sensitivity but comparable specificity to correctly identify HSIL histological lesions. However, using a cut-point of ASCUS, differences in accuracy were of borderline significance (cervical vs. anal: p = 0.04; I^2^ = 68). Study heterogeneity was large in both screening settings. Funnel plots (Figure 3), demonstrate that relatively more of the cervical screening studies fall outside the pseudo 95% confidence intervals than is observed for the anal screening studies, however, the relative symmetry of both funnel plots is supported by the non-significant Egger test for both. Figure 4 presents ROC curves based on alternative cytology cut-points and compares the ability of anal and cervical cytology to discriminate between histopathologic categories (<HSIL vs. ≥ HSIL).

## Discussion

Anal cancer screening has not been recommended as standard of care in HIV clinics in the United States with the exception of the State of New York [51]. Our cytology cut-point specific meta-analytic comparison of the relative accuracy of cervical and anal cytology allows the following conclusions to be made: (1) anal cytology is overall less discriminating than cervical cytology using ROC area as the metric of test discrimination; (2) if a cytology cut-point of NAMC vs. ≥ ASCUS is used, the sensitivity in both settings is nearly identical while anal cytology is meaningfully less specific than cervical cytology; (3)at cytology cut-point of (HSIL or ASC-H) vs. ≤ LSIL, specificity of anal and cervical cytology is nearly identical while sensitivity of anal cytology is meaningfully less than that of cervical cytology. In the setting of screening for anal cancer and its potentially modifiable precursor lesions, we believe that a selecting a cytology cut-point with maximal sensitivity is preferable to selecting a cut-point that maximizes specificity. The primary reason we argue this position is that, as we have previously shown [52], neither cervical nor anal histopathology obtained by colposcope magnified punch biopsy is a true “gold standard”. Punch biopsy in both settings is subject to several sources of error including sampling error, operator error, and interpretation error [53], [54]. Thus many false positive cytology results may in fact be erroneously classified because of the fallibility of the reference standard punch biopsy.

In summary, using a cytology cut-point of HSIL or ASC-H, anal cytology is less sensitive but comparably specific compared to cervical cytology. However, using a cut-point of ASCUS, differences in accuracy were of borderline significance. These results contribute to the evidence base regarding screening accuracy and might inform discussion regarding potential guidelines for screening for anal cancer and its precursors in high risk populations.

## Supporting Information

Table S1 **Extracted Study Data and Outcome Metrics, by Study Type Cytology-Biopsy Joint Cell Frequencies.**
(DOC)
